# Mechanical Ventilation Induces an Inflammatory Response in Preinjured Lungs in Late Phase of Sepsis

**DOI:** 10.1155/2015/364020

**Published:** 2015-06-16

**Authors:** Wei Xuan, Quanjun Zhou, Shanglong Yao, Qingzhu Deng, Tingting Wang, Qingping Wu

**Affiliations:** Department of Anesthesiology, Union Hospital, Tongji Medical College, Huazhong University of Science and Technology, Wuhan 430022, China

## Abstract

Mechanical ventilation (MV) may amplify the lung-specific inflammatory response in preinjured lungs by elevating cytokine release and augmenting damage to the alveolar integrity. In this study, we test the hypothesis that MV exerts different negative impacts on inflammatory response at different time points of postlung injury. Basic lung injury was induced by cecal ligation and puncture (CLP) surgery in rats. Physiological indexes including blood gases were monitored during MV and samples were assessed following each experiment. Low *V*_*T*_ (tidal volume) MV caused a slight increase in cytokine release and tissue damage at day 1 and day 4 after sepsis induced lung injury, while cytokine release from the lungs in the two moderately ventilated *V*_*T*_ groups was amplified. Interestingly, in the two groups where rats received low *V*_*T*_ MV, we found that infiltration of inflammatory cells was only profound at day 4 after CLP. Marked elevation of protein leakage indicated a compromise in alveolar integrity in rats that received moderate *V*_*T*_ MV at day 4 following CLP, correlating with architectural damage to the alveoli. Our study indicates that preinjured lungs are more sensitive to mechanical MV at later phases of sepsis, and this situation may be a result of differing immune status.

## 1. Introduction

Patients suffering from acute lung injury (ALI) or acute aspiratory distress syndrome (ARDS) are likely to receive mechanical ventilation (MV) treatment as a therapeutic intervention [1]. Although MV is necessary and life-saving, it may cause lung injury or exacerbate preexisting lung injury, a condition known as ventilator-associated lung injury (VILI) [2, 3]. Curative strategy of MV can cause VILI via the induction of oxidant stress and neutrophil infiltration in a rat model [4].

Sepsis is a critical state of inflammation with high morbidity and mortality rates in the intensive care unit (ICU) [5]. Certain factors, such as overgeneration of reactive oxygen species (ROS), play important roles between sepsis and VILI. Both in vivo and in vitro studies have demonstrated that oxidative stress, plus dysfunction of antioxidant system, leads to the onset or deterioration of ALI after sepsis and VILI [6, 7]. On the other hand, sepsis can overwhelm the body resulting in immune suppression, leaving patients more susceptible to secondary infections due to an inability to mount an effective inflammatory response [8–10]. The generation of reactive oxygen species by immune cells can be changed depending on different phase of sepsis [11], in which persistence indicates a poor result and may affect the outcome of VILI due to the modulation of ROS elimination. Previous studies have shown that MV had a negative impact on preinjured lungs or other organs affected by sepsis [12–14].

The purpose of this study was to investigate how MV impacts upon preinjured lung function at different time points after sepsis induction. We used a clinically relevant septic rat model to assess prolonged lung injury. We hypothesized that the negative impacts of MV on preinjured lung at the later phase of sepsis may be more severe than those observed in the early phase. We did not include a high *V*_*T*_ (more than 12 mL/kg) of MV in our current study due to the fact that it was not clinically relevant and this method results in *V*_*T*_-induced serious lung injury in patients and a range of animal models [15–17]. To evaluate the effects of MV on preexisting lung injury, we examined the physiological response, lung injury, inflammatory cell accumulation and infiltration, and cytokine release using septic rat model.

## 2. Materials and Methods

### 2.1. Animals and Experimental Sepsis

We used pathogen-free male Sprague-Dawley rats (weighing 250–300 g). All rats in the sepsis groups received cecal ligation and puncture (CLP) surgery. In brief, rats were fasted 16 h before surgery and then anesthetized by an injection of 1 mL of 2% pentobarbital in the peritoneal cavity. Under sterile conditions, a 2 cm incision was made on the midline of the abdomen, before exposing the cecum. The cecum was ligated one-third along its distal position and punctured twice with a 16-gauge needle. This operation caused ALI and had a 95% survival rate in our preliminary study (data not shown). The cecum was returned to the peritoneal cavity and the incision was closed in two layers by suture. All the animals received subcutaneous application of 10 mL/kg sterile saline for fluid resuscitation. The experimental protocol was approved by the Animal Care and Scientific Committee of Tongji Medical College, Huazhong University of Science and Technology.

### 2.2. Mechanical Ventilation (MV) Protocol

All rats were randomly allocated into seven groups as follows (four MV groups were ventilated for 4 hours with room air): control group: control rats (*n* = 6); group CLP1day: septic rats were sacrificed at day 1 after CLP without MV (*n* = 6); group CLP1day + LMV: septic rats received MV at day 1 after CLP, low *V*_*T*_ (6 mL/kg), 4 cm H_2_O ZEEP (*n* = 8); group CLP1day + MMV: septic rats received MV at day 1 after CLP, moderate *V*_*T*_ (12 mL/kg), 2 cm H_2_O ZEEP (*n* = 8); group CLP4day: septic rats were sacrificed at day 4 after CLP without MV (*n* = 6); group CLP4day + LMV: septic rats received MV at day 4 after CLP, low *V*_*T*_ (6 mL/kg), 4 cm H_2_O ZEEP (*n* = 8); group CLP4day + MMV: septic rats received MV at day 4 after CLP, moderate *V*_*T*_ (12 mL/kg), 2 cm H_2_O ZEEP (*n* = 8). All rats were anesthetized by intraperitoneal injection of 2% pentobarbital (1 mL) before undergoing tracheotomy and were connected to a small-animal ventilator (Harvard Apparatus, Holliston, MA, USA). Anesthesia was maintained by constant injection of pentobarbital (80 mg/kg/h) and fluids were administered at a rate of 10 mL/kg/h by jugular vein intubation. A catheter was inserted into the left carotid artery for blood pressure measurements and blood gas analysis every two hours. We kept partial pressure of arterial carbon dioxide (*P*_*a*_CO_2_) between 35 and 45 mmHg by changing breathing rate. Body temperature was monitored rectally and regulated automatically by a heating pad. All animals were sacrificed by abdominal aorta exsanguination at experiment completion.

### 2.3. Histology of the Lung

The superior lobe of the right lung was fixed in 10% pH neutral formalin for 24 h, cut into 10 *µ*m sections, and mounted on glass slides. The tissue sections were stained with hematoxylin and eosin (H&E) and examined by light microscopy. The histological alterations were scored in five grades from 0 to 4 depending on the degree of microscopic damage of architectural integrity, inflammatory cell infiltration, interstitial edema, and hemorrhage.

### 2.4. Cell Counts

Lung lavages were performed with 3 × 2 mL cold sterile PBS; the fluid was centrifuged at 150 ×g at 4°C for 10 min. After centrifugation, the supernatant was collected and frozen at −70°C for further analysis. The cell pellet was resuspended in 0.5 mL cold PBS. Total cell counts of bronchoalveolar lavage fluid (BALF) were determined using a cell counting chamber. Slides of the differential counts were performed using a cytocentrifuge at 150 ×g for 5 min, followed by Wright staining.

### 2.5. Cytokine Assays

Level of interleukin- (IL-) 6 in the supernatant of BALF was measured by enzyme-linked immunosorbent assay (ELISA) according to the manufacturer's protocol. Briefly, samples or standards were pipetted into microplate wells precoated with monoclonal antibody. After washing away unbound substances, an enzyme-linked polyclonal antibody specific for rat IL-6 (BD Biosciences Pharmingen, San Diego, CA, USA) was added to the microplate wells. Following a wash to remove any unbound antibody-enzyme reagent, substrate solution was added to the wells. The enzyme reaction yielded a blue product that turned yellow when the phosphoric acid stop solution was added. The intensity of the color was proportionate to the amount of total rat IL-6 bound in the initial step and the sample values were then extrapolated from the standard curve. Total protein levels in BALF were determined by Pierce BCA Protein Assay kit (Thermo Scientific, Rockford, IL, USA) according to the manufacturer's instructions with bovine serum albumin (BSA) as a standard.

### 2.6. Statistical Analysis

Data are expressed as mean ± SD (standard deviation). All parameters were analyzed by one-way analysis of variance (ANOVA) with least significant difference (LSD) posttest. The histological injury scores comparisons were made by the nonparametric Mann-Whitney test. *P* values of <0.05 were considered to be statistically significant.

## 3. Results

Two rats died from cardiovascular collapse: one in group two and one in group 3. Therefore, only 48 rats out of 50 were studied.

### 3.1. MV Impacts on Physical Signs

**Figure 1:**
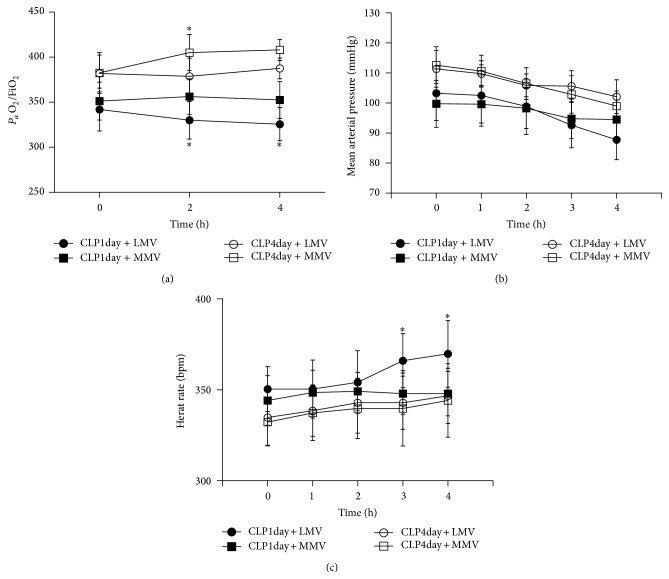
Physiological response to mechanical ventilation following induction of sepsis over time. *P*_*a*_O_2_/FiO_2_ (a), mean arterial pressure (b), and heart rate (c) were measured. *P* values are calculated by repeated-measures ANOVA. Significant differences are highlighted between groups receiving MV on the same day. ^*^The same time point at which the group received MV at the same day after CLP. Data are presented as mean ± SD.

There was no statistically significant difference in *P*_*a*_O_2_/FiO_2_ among the four MV groups at the start of ventilation. Furthermore, the oxygen index (OI) was only slightly higher in the two CLP day-four groups (Figure 1(a)). The OI differed over time in all groups with the lowest *P*_*a*_O_2_/FiO_2_ occurring in group CLP1day + LMV, while the highest OI occurred in group CLP4day + MMV.

The initial mean arterial pressure (MAP) was similar and gradually decreased over time in all groups (Figure 1(b)). The lowest blood pressure measurement was observed in group CLP1day + LMV, but this drop was not statistically significant. Heart rate (HR) was steady in the other three MV groups but had a statistically significant rise in group CLP1day + LMV after 2 h MV (Figure 1(c)).

Although there was a trend of lower pH in groups CLP1day + LMV and CLP1day + MMV, the pH values generally matched those in groups CLP4day + LMV and CLP4day + MMV (data not shown).

### 3.2. Lung Injury

**Figure 2:**
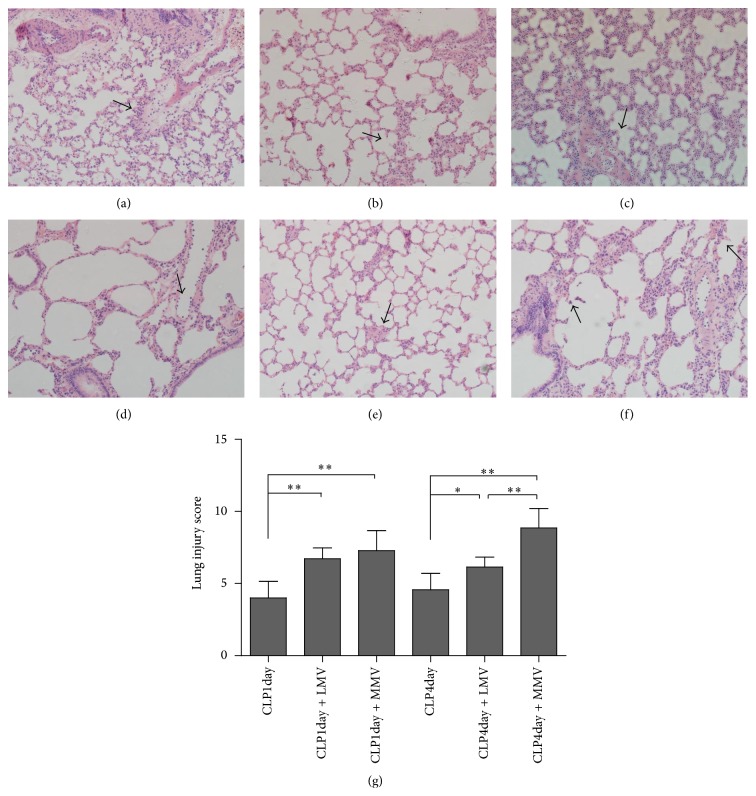
Representative hematoxylin and eosin-stained 5 *µ*m lung sections at 400x magnification. CLP at day one without MV (a), with low *V*_*T*_ MV (b), and with moderate *V*_*T*_ MV (c). CLP at day four without MV (d), with low *V*_*T*_ MV (e), and with moderate *V*_*T*_ MV (f) are shown. Lung injuries according to the scoring system are pointed with black arrow in the figures. Lung injury score is presented as bar chart on the right (g). Data are presented as mean ± SD.

CLP surgery caused moderate structural damage of lung, slight cellular infiltration, and edema at days one and four after operation (Figures 2(a) and 2(d)). We saw more severe accumulation of inflammatory cells in groups CLP1day + LMV and CLP4day + LMV (Figures 2(b) and 2(e)). For moderate *V*_*T*_ MV one day after sepsis, these histological changes were similar to those in two low *V*_*T*_ groups (Figure 2(c)). The impacts of MV were most evident in group CLP4day + MMV, with partial interstitial edema and areas of alveolar hemorrhage (Figure 2(f)), which showed that relatively higher tidal volume MV exerted more negative impact on preinjured lung at day four after CLP compared with day one following CLP.

### 3.3. Cell Counts

**Figure 3:**
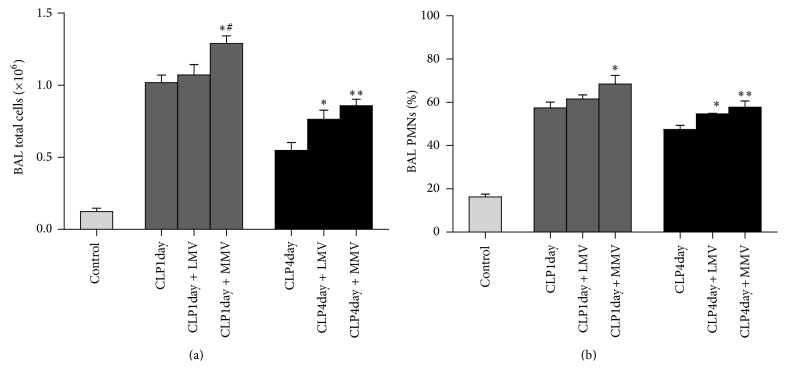
Total cell counts (a) and polymorphonuclear cell count (PMN) (b) in BAL for each of the seven groups. ^*∗*^Without MV at the same day after CLP; ^#^low *V*_*T*_ MV at the same day after CLP. Data are presented as mean ± SD.

**Figure 4:**
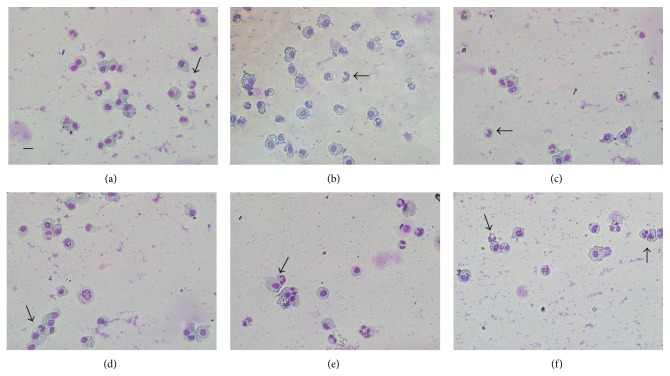
Differential analysis of PMNs is presented at 400x magnification. CLP at day one without MV (a), with low *V*_*T*_ MV (b), and with moderate *V*_*T*_ MV (c). CLP at day four without MV (d), with low *V*_*T*_ MV (e), and with moderate *V*_*T*_ MV (f). Polymorphonuclear neutrophils are pointed with black arrow. Bar = 50 *µ*m.

BALF total cell counts are an important indicator of the integrity of lung capillary. From the results, we can see that CLP surgery increased the total cell counts in BALF (Figure 3(a)). There were large increases in groups of CLP4day + LMV and CLP4day + MMV, while in the groups of day one after CLP, only MMV caused a significant increase of total cell counts. Differential analysis revealed similar increases of polymorphonuclear (PMN) cells in BALF except in group CLP1day + LMV (Figures 3(b) and 4). Examination of tissue slides under light microscopy validated these observations. Additionally, MV magnified the inflammatory cells infiltration to a deeper extent at day four after CLP compared with day one after CLP. These data are in accordance with the findings from histological sections.

### 3.4. Total Protein and IL-6 Levels

**Figure 5:**
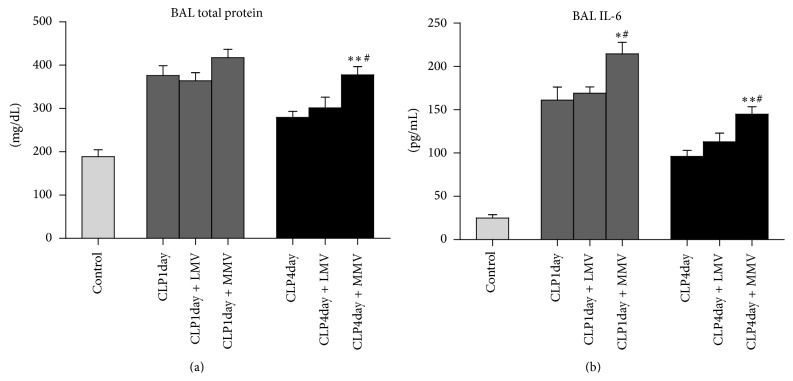
Total protein (a) and IL-6 (b) in BAL for each of the seven groups. ^*∗*^Without MV at the same day after CLP; ^#^low *V*_*T*_ MV at the same day after CLP. Data are presented as mean ± SD.

BALF total protein level was quantified to identify the status of capillary permeability and IL-6 was selected as a representative inflammatory cytokine. Studies have shown that moderate *V*_*T*_ can augment rabbit lung cytokine release, such as TNF-*α* and IL-8, after the systemic of systemic LPS [18]. IL-6 is regarded as an active factor and an ongoing inflammatory marker of many lung diseases [19]. The synergistic role of IL-6 in pathologic mechanical stretch induced lung endothelial cell injury has also been demonstrated in vitro [20]. As shown in Figure 5, both total protein and IL-6 were significantly higher in group CLP4day + MMV when compared to groups CLP4day and CLP4day + LMV, which indicated that, on the fourth day after initiation of CLP, relatively higher volume of MV severely increases both lung capillary permeability and cytokine release. Furthermore, IL-6 was significantly higher in group CLP1day + MMV compared with groups CLP1day and CLP1day + LMV, while there were no statistically significant differences in total levels of protein observed between these groups (Figure 5). These results suggested that, at the early stage of sepsis (day one after CLP), when compared to low tidal volume, moderate tidal volume of MV changed inflammatory cytokine release but had no effect on lung capillary permeability.

## 4. Discussion

The aim of this study was to investigate whether MV exerts distinct effects on lungs at different time after lung injury. We hypothesized that MV would induce an inflammatory response following induction of sepsis by CLP surgery and that this response would be amplified over time. According to our findings, no changes could be found in the inflammatory response at one day after sepsis induction by LMV, but both LMV and MMV did show more severe inflammatory cytokine release and lung injury at a later phase of sepsis.

Acute lung injury (ALI) is a common life-threatening condition that often needs the treatment of MV [1]. Nevertheless, MV can amplify the inflammatory response of preinjured lungs or induce massive release of cytokines from healthy lungs [21, 22], a finding that was validated in our current study. In patients with ARDS, higher tidal volume of MV increases mortality [23]. A recent study showed that MV with a low tidal volume (6 mL/kg) reduced the incidence of mechanical associated lung injury in patients without preexisting lung injury [24]. Therefore, it is now a common view among experts that MV with a low tidal volume protects lungs for patients with and without preexisting lung injury. However, no studies have investigated whether preinjured lungs responded differently to MV at different periods of sepsis. We believe that this information will be meaningful for the determination of the proper tidal volume on a case-by-case basis.

Negative synergistic effects of MV at single time points after sepsis have been found and higher tidal volume tends to be more detrimental [16, 25, 26]. We examined whether moderate tidal volume could be more detrimental to preinjured lungs when compared to low tidal volume in two typical phases of sepsis (one day versus four days) in this study. Furthermore, we examined the effects of two settings (low versus moderate tidal volume) of MV on lungs at different days after initiation of lung injury induced by sepsis.

Low *V*_*T*_ MV caused some heart rate fluctuation and insufficient oxygen index in the first day in the CLP group, where early phase of sepsis was used. Moderate tidal volume MV provided better oxygenation and hemodynamic stability. We found that BALF total protein concentrations were not significantly different among the groups except on the fourth day with moderate MV, which caused a significant increase in total protein concentration. This could be explained by the more severe histological alterations observed in the same group, in which the loss of alveolar integrity would cause more protein leakage.

In terms of BALF total cell counts and PMN differential counts, we observed that effects of moderate *V*_*T*_ MV were similar at different days following sepsis. However, low *V*_*T*_ MV attracted more inflammatory cells and induced more PMN differentiation at the fourth day after sepsis. The concentrations of IL-6 were greater in the group receiving moderate *V*_*T*_ MV at the first and fourth day. This trend was more evident in group 7 on the fourth day after CLP.

These results suggest that although there are some differences between low and moderate *V*_*T*_ MV at the first day of sepsis in our model, moderate *V*_*T*_ MV four days after sepsis are more likely to exert a negative impact on inflammatory lungs responses. Furthermore, for rats receiving low *V*_*T*_ MV, only after four days were synergistic effects on total cell and PMN differential counts observed. This consequence may result from diverse functions of immune cells during sepsis. The immune system plays an important role in the host-pathogen relationship, particularly during sepsis, since sepsis can cause immune suppression that affects the response to other infectious threats [27]. Patients suffering from sepsis undergo certain adaptive responses including a phase of immunosuppression [28–30], and animals are susceptible to infections in the lung or peripheral blood [31, 32]. This situation may be induced by impaired function of immune cells or dysfunctional cell signaling pathways [10, 33, 34]. Since MV can influence immune cells such as macrophages or alveolar epithelial cells to cause trauma [35, 36], different immune cell states determine different influences of MV. Furthermore, due to the influence of a number of perioperative factors, certain cell functions can be modulated via ROS system [37]. The expression of toll-like receptors (TLRs) is also associated with mechanical induction of lung injury [38, 39]. Previous studies have shown that signal transduction of TLRs can be modulated by sepsis [10]. Taking this into consideration, TLR4 may directly alter the interaction between MV and lungs that were preinjured by sepsis. At the early phase of sepsis, immune cells may not be sensitive to MV due to immunosuppression; thus MV only cause mild inflammatory response.

The function of immune system and ROS generation are variable at a different time point during the process of sepsis [12]. Campos et al. revealed that antioxidant* N*-acetylcysteine could alleviate ALI after CLP followed by low *V*_*T*_ MV in a rat, and this protective effect was attributed to the decrease of oxidative stress [13]. ROS also has the property to increase TLR expression of alveolar macrophages in a mice hemorrhagic shock model [40]. Based on the interaction between MV and immune cells mentioned above, it is reasonable to link the variation of VILI to the production of ROS that at the later phase of sepsis, the fourth day after CLP. In our current experiment, more severe lung injury can be found after the treatment of MV.

Our results confirm that although lung-protective MV with low *V*_*T*_ is important in minimizing the negative impact of MV on lungs, sometimes it cannot meet all physiological requirements in the septic rat model. It may be beneficial to increase the tidal volume in certain periods after sepsis to improve the oxygenation or stabilize the circulation system, without worrying about the consequent inflammatory response. However, our study suggests that a specific window of time exists at the later phase of sepsis to consider the changes to MV as an intervention.

## 5. Conclusions

We have found that moderate *V*_*T*_ MV causes a more severe inflammatory response when compared to low *V*_*T*_ MV in preinjured lungs, and this effect is more evident at a later phase (fourth day) of sepsis. Furthermore, low *V*_*T*_ MV causes greater synergistic inflammatory effects at later period of sepsis. It will be important to investigate if the different status of ROS generation of immune cells contributes to these phenomena in future studies.

## References

[1] Matthay M. A., Zemans R. L. (2011). The acute respiratory distress syndrome: pathogenesis and treatment. *Annual Review of Pathology: Mechanisms of Disease*.

[2] Dreyfuss D., Saumon G. (1998). Ventilator-induced lung injury: lessons from experimental studies. *The American Journal of Respiratory and Critical Care Medicine*.

[3] Frank J. A., Parsons P. E., Matthay M. A. (2006). Pathogenetic significance of biological markers of ventilator-associated lung injury in experimental and clinical studies. *Chest*.

[4] Li L.-F., Yang C.-T., Huang C.-C., Liu Y.-Y., Kao K.-C., Lin H.-C. (2011). Low-molecular-weight heparin reduces hyperoxia-augmented ventilator-induced lung injury via serine/threonine kinase-protein kinase B. *Respiratory Research*.

[5] Martin G. S., Mannino D. M., Eaton S., Moss M. (2003). The epidemiology of sepsis in the United States from 1979 through 2000. *The New England Journal of Medicine*.

[6] Tasaka S., Amaya F., Hashimoto S., Ishizaka A. (2008). Roles of oxidants and redox signaling in the pathogenesis of acute respiratory distress syndrome. *Antioxidants and Redox Signaling*.

[7] Chapman K. E., Sinclair S. E., Zhuang D., Hassid A., Desai L. P., Waters C. M. (2005). Cyclic mechanical strain increases reactive oxygen species production in pulmonary epithelial cells. *American Journal of Physiology—Lung Cellular and Molecular Physiology*.

[8] Wynn J., Cornell T. T., Wong H. R., Shanley T. P., Wheeler D. S. (2010). The host response to sepsis and developmental impact. *Pediatrics*.

[9] Cohen J. (2002). The immunopathogenesis of sepsis. *Nature*.

[10] Deng J. C., Cheng G., Newstead M. W. (2006). Sepsis-induced suppression of lung innate immunity is mediated by IRAK-M. *The Journal of Clinical Investigation*.

[11] Santos S. S., Brunialti M. K. C., Rigato O., MacHado F. R., Silva E., Salomao R. (2012). Generation of nitric oxide and reactive oxygen species by neutrophils and monocytes from septic patients and association with outcomes. *Shock*.

[12] Herrera M. T., Toledo C., Valladares F. (2003). Positive end-expiratory pressure modulates local and systemic inflammatory responses in a sepsis-induced lung injury model. *Intensive Care Medicine*.

[13] Campos R., Shimizu M. H. M., Volpini R. A. (2012). N-acetylcysteine prevents pulmonary edema and acute kidney injury in rats with sepsis submitted to mechanical ventilation. *The American Journal of Physiology—Lung Cellular and Molecular Physiology*.

[14] Kneyber M. C. J., Gazendam R. P., Niessen H. W. M. (2009). Mechanical ventilation during experimental sepsis increases deposition of advanced glycation end products and myocardial inflammation. *Critical Care*.

[15] Lellouche F., Dionne S., Simard S., Bussières J., Dagenais F. (2012). High tidal volumes in mechanically ventilated patients increase organ dysfunction after cardiac surgery. *Anesthesiology*.

[16] Nin N., Lorente J. A., Fernández-Segoviano P., De Paula M., Ferruelo A., Esteban A. (2009). High-tidal volume ventilation aggravates sepsis-induced multiorgan dysfunction in a dexamethasone-inhibitable manner. *Shock*.

[17] Menendez C., Martinez-Caro L., Moreno L. (2013). Pulmonary vascular dysfunction induced by high tidal volume mechanical ventilation. *Critical Care Medicine*.

[18] Altemeier W. A., Matute-Bello G., Frevert C. W. (2004). Mechanical ventilation with moderate tidal volumes synergistically increases lung cytokine response to systemic endotoxin. *American Journal of Physiology: Lung Cellular and Molecular Physiology*.

[19] Rincon M., Irvin C. G. (2012). Role of IL-6 in asthma and other inflammatory pulmonary diseases. *International Journal of Biological Sciences*.

[20] Birukova A. A., Tian Y., Meliton A., Leff A., Wu T., Birukov K. G. (2012). Stimulation of Rho signaling by pathologic mechanical stretch is a “second hit” to Rho-independent lung injury induced by IL-6. *American Journal of Physiology: Lung Cellular and Molecular Physiology*.

[21] Whitehead T. C., Zhang H., Mullen B., Slutsky A. S. (2004). Effect of mechanical ventilation on cytokine response to intratracheal lipopolysaccharide. *Anesthesiology*.

[22] Vaneker M., Halbertsma F. J., van Egmond J. (2007). Mechanical ventilation in healthy mice induces reversible pulmonary and systemic cytokine elevation with preserved alveolar integrity: an in vivo model using clinical relevant ventilation settings. *Anesthesiology*.

[23] Brower R. G., Matthay M. A., Morris A., Schoenfeld D., Thompson B. T., Wheeler A. (2000). Ventilation with lower tidal volumes as compared with traditional tidal volumes for acute lung injury and the acute respiratory distress syndrome. *The New England Journal of Medicine*.

[24] Determann R. M., Royakkers A., Wolthuis E. K. (2010). Ventilation with lower tidal volumes as compared with conventional tidal volumes for patients without acute lung injury: a preventive randomized controlled trial. *Critical Care*.

[25] Villar J., Cabrera N., Casula M. (2010). Mechanical ventilation modulates Toll-like receptor signaling pathway in a sepsis-induced lung injury model. *Intensive Care Medicine*.

[26] Nakamura T., Malloy J., McCaig L. (2001). Mechanical ventilation of isolated septic rat lungs: effects on surfactant and inflammatory cytokines. *Journal of Applied Physiology*.

[27] van der Poll T., Opal S. M. (2008). Host-pathogen interactions in sepsis. *The Lancet Infectious Diseases*.

[28] Gorbunov N. V., Garrison B. R., McDaniel D. P. (2013). Adaptive redox response of mesenchymal stromal cells to stimulation with lipopolysaccharide inflammagen: mechanisms of remodeling of tissue barriers in sepsis. *Oxidative Medicine and Cellular Longevity*.

[29] Volk H. D., Reinke P., Docke W. D. (2000). Clinical aspects: from systemic inflammation to ‘immunoparalysis’. *Cd14 in the Inflammatory Response*.

[30] Hotchkiss R. S., Nicholson D. W. (2006). Apoptosis and caspases regulate death and inflammation in sepsis. *Nature Reviews Immunology*.

[31] Davis C. G., Chang K., Osborne D., Walton A. H., Dunne W. M., Muenzer J. T. (2011). Increased susceptibility to Candida infection following cecal ligation and puncture. *Biochemical and Biophysical Research Communications*.

[32] Benjamim C. F., Hogaboam C. M., Lukacs N. W., Kunkel S. L. (2003). Septic mice are susceptible to pulmonary aspergillosis. *The American Journal of Pathology*.

[33] Coelho A. L., Schaller M. A., Benjamim C. F., Orlofsky A. Z., Hogaboam C. M., Kunkel S. L. (2007). The chemokine CCL6 promotes innate immunity via immune cell activation and recruitment. *Journal of Immunology*.

[34] Benjamim C. F., Lundy S. K., Lukacs N. W., Hogaboam C. M., Kunkel S. L. (2005). Reversal of long-term sepsis-induced immunosuppression by dendritic cells. *Blood*.

[35] Kuipers M. T., Aslami H., Janczy J. R. (2012). Ventilator-induced lung injury is mediated by the NLRP3 inflammasome. *Anesthesiology*.

[36] Cohen T. S., Lawrence G. G., Khasgiwala A., Margulies S. S. (2010). MAPk activation modulates permeability of isolated rat alveolar epithelial cell monolayers following cyclic stretch. *PLoS ONE*.

[37] Tang J., Hu J. J., Lu C. H. (2014). Propofol inhibits lipopolysaccharide-induced tumor necrosis factor-alpha expression and myocardial depression through decreasing the generation of superoxide anion in cardiomyocytes. *Oxidative Medicine and Cellular Longevity*.

[38] Vaneker M., Joosten L. A., Heunks L. M. A. (2008). Low-tidal-volume mechanical ventilation induces a toll-like receptor 4-dependent inflammatory response in healthy mice. *Anesthesiology*.

[39] Charles P. E., Barbar S. D. (2010). Toll-like receptors: a link between mechanical ventilation, innate immunity and lung injury?. *Intensive Care Medicine*.

[40] Fan J., Li Y., Vodovotz Y., Billiar T. R., Wilson M. A. (2007). Neutrophil NAD(P)H oxidase is required for hemorrhagic shock-enhanced TLR2 up-regulation in alveolar macrophages in response to LPS. *Shock*.

